# Insights into the molecular changes of adipocyte dedifferentiation and its future research opportunities

**DOI:** 10.1016/j.jlr.2024.100644

**Published:** 2024-09-18

**Authors:** Mingheng Xue, Yunjun Liao, Wenqing Jiang

**Affiliations:** Department of Plastic and Cosmetic Surgery, Nanfang Hospital, Southern Medical University, Guangzhou, Guangdong, China

**Keywords:** cell therapy, dedifferentiated fat cell, lipid metabolism, redifferentiation, regenerative medicine

## Abstract

Recent studies have challenged the traditional belief that mature fat cells are irreversibly differentiated and revealed they can dedifferentiate into fibroblast-like cells known as dedifferentiated fat (DFAT) cells. Resembling pluripotent stem cells, DFAT cells hold great potential as a cell source for stem cell therapy. However, there is limited understanding of the specific changes that occur following adipocyte dedifferentiation and the detailed regulation of this process. This review explores the epigenetic, genetic, and phenotypic alterations associated with DFAT cell dedifferentiation, identifies potential targets for clinical regulation and discusses the current applications and challenges in the field of DFAT cell research.

## Graphical Abstract

**Figure:**
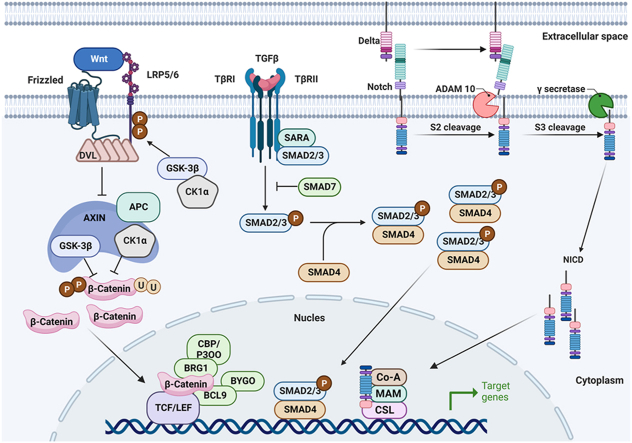

Adipose tissue is the largest endocrine organ in the human body, consisting primarily of a vast number of mature fat cells responsible for storing, utilizing, and releasing lipids (1). In addition to these functions, fat cells release various hormones, growth factors, and cytokines that play pivotal roles in the local niche as well as other tissues and organs. The dual roles of adipose tissue in energy regulation and endocrine function establishes it as a crucial organ for maintaining metabolic homeostasis of the entire body (1).

Traditionally, mature fat cells with large lipid droplets (LDs) have been viewed as an irreversible and terminal stage of differentiation. However, recent studies have challenged this perspective by demonstrating that mature fat cells can dedifferentiate to acquire a fibroblast-like morphology, which is often referred to as fat fibroblasts or dedifferentiated fat (DFAT) cells (2, 3, 4, 5). DFAT cells share similarities with fibroblasts in terms of morphology but exhibit functional characteristics akin to pluripotent stem cells, particularly their ability to differentiate into multiple cell lineages (2, 3, 4, 5). This capacity enables DFAT cells as a promising cell source for regenerative medicine.

Dedifferentiation of adipocytes involves restoration of their morphology from a large, unilocular state to a fibroblast-like state (3). This process reduces both the size and number of adipocytes, potentially offering a strategy to address issues of obesity and localized fat accumulation. Moreover, DFAT cells have been identified in many in vivo pathological conditions, such as breast cancer and pancreatic cancer, associated with tumor progression (6, 7) and are not restricted to in vitro induction alone. Therefore, precise regulation of adipocyte dedifferentiation holds significant clinical promise for applications in regenerative medicine and disease treatment.

This article reviews the epigenetic, genetic, and phenotypic changes that occur in the post-dedifferentiation phase of DFAT cells, discusses potential targets for dedifferentiation regulation, explores clinical applications, and highlights the current challenges for DFAT cells.

## Changes Associated with Adipocyte Dedifferentiation

### Adipocyte dedifferentiation in vitro and in vivo

The large size, low density, and high lipid content of adipocytes make it very difficult to culture primary adipocytes in vitro. In 1986, Sugihara *et al.* first reported a dedifferentiation method for DFAT cells derived from mature fat cells, commonly known as the ceiling culture technique (3). This method involves culturing fat cells in a bottle filled with medium, where buoyancy leads the fat cells to attach to the top of the bottle. Over the next 7–10 days, the fat droplets within the fat cells gradually disappeared, and the unilocular fat cells transformed into fibroblast-like forms (3). Recently, in vitro studies have demonstrated that DFAT cells can be obtained from subcutaneous white adipose tissue (WAT) of various species, including humans (3, 8), rats (9), mice (10), and bovines (11). Regarding the dedifferentiation of different types of fat cells, other studies have shown that mouse brown fat cells can dedifferentiate under tumor necrosis factor (TNF)—α stimulation, which demonstrated that brown fat cells possess similar plasticity as white fat cells (12). Direct evidence for the dedifferentiation of beige fat cells is rarely reported, possibly due to its dearth of reliable in vitro models. However, a recent study reported a new method for the conversion of beige adipocytes using the ceiling culture technique (13), offering hope for further research on their plasticity.

Adipocyte dedifferentiation can also be induced by various in vitro stimuli. Recent studies have used inducers such as Wnt3a (14), transforming growth factor-β (TGF-β1) (15), the insulin signaling blocker OSI-906 (16), TNF-α (17), found in inflammatory zone 1 (FIZZ1) (18), and oncostatin M (OSM) (19) to promote dedifferentiation of mature adipocytes. Li *et al.* found that high osmotic pressure (400 mmol), mimicking the tumor microenvironment, causes adipocytes to gradually lose LDs, adopting a fibroblast-like morphology and regaining multipotent differentiation potential (20). Additional studies have shown that adipocytes exposed to regular cold stimulation (10°C) have smaller LDs and reduced lipid contents and can dedifferentiate into fibroblast-like cells (21).

More importantly, DFAT cells are not merely a simple cell culture phenomenon. Adipocyte dedifferentiation has been observed in various physiological and pathological in vivo environments. A recent study using AdipoChaser-LacZ mouse models tracked the fate of adiponectin-expressing adipocytes in the mammary gland during pregnancy, lactation, and aging. It was found that fat cells in the breast underwent dedifferentiation during pregnancy and lactation, becoming fibroblast-like cells that resided between breast alveolar structures. Single-cell RNA sequencing revealed that the expression of common Adipoq markers (*Adipoq*, *Fabp4*, *Pparg*, etc.) in these dedifferentiated adipocytes was significantly lower compared to mature mammary adipocytes. The expression of common preadipocyte markers (*Pdgfra*, *Pdgfrb*, etc.) and common fibroblast markers (*Vim*, *Col1a1*, etc.) was recovered. After lactation ceased, these dedifferentiated cells could proliferate and redifferentiate into mature fat cells, which recurred during at least two pregnancies (22, 23). Dedifferentiation of dermal adipocytes also plays an important role in the hair cycle. Dermal fat cells undergo significant changes at different stages of the hair growth cycle, expanding during the growth phase and significantly decreasing in volume during the regression and rest phases. During the degenerative phase of hair growth, mature dermal fat cells dedifferentiate into a preadipocyte or fibroblast-like state, accompanied by the reexpression of Pdgfrα and other preadipocyte marker genes. These dedifferentiated cells can subsequently be stimulated by growth signals to redifferentiate into mature fat cells, thus participating in various stages of hair growth (24, 25).

Adipocyte dedifferentiation has also been observed in pathological conditions such as skin fibrosis, wound healing, and cancer using lineage tracing techniques in mouse models (6, 26, 27). In an AdipoP-Cre transgenic mouse model of bleomycin-induced skin fibrosis, triple-positive "transition cells" expressing tdTomato, perilipin (a marker typical of adipocytes), and α-SMA (a marker typical of myofibroblasts) were observed in dermal white adipose tissue (dWAT), followed by a loss of perilipin expression during fibrosis accumulation, indicating the transformation of adipocytes into myofibroblasts (26). Other studies have found that fat cells in dWAT undergo lipolysis due to skin injury during the healing process. Extensive lipolysis depletes the stored lipids in the cells, allowing dermal fat cells to dedifferentiate into fibroblasts, migrate to the wound bed, and subsequently redifferentiate into fat cells to accelerate wound healing (27). A recent study simulated liposarcoma (LPS) in a mouse model of adipocyte-specific activation of Notch signaling (Ad/N1ICD). It found that Ad/N1ICD adipocytes underwent dedifferentiation before transforming into LPS, resulting in lipodystrophy and metabolic dysfunction, which contributed to the occurrence and development of LPS (28). Additional studies have shown that 3T3-L1 adipocytes cocultured with pancreatic cancer cells (MiaPaCa2) or melanoma cells lose fat droplets and dedifferentiate into cancer-associated adipocytes, which in turn contribute to malignant features of tumors (7, 29, 30).

Due to the lack of specific cell surface markers in DFAT cells when compared to adipose-derived stem cells (ASCs), it is very difficult to directly observe DFAT cells from human adipose tissue samples. However, a recent study, in which human mature adipocytes labeled with PKH26 were injected into nude mice, found that the grafted adipocytes exhibited positive expression of CD90 and CD105 and negative expression of CD45 on the surface of PKH26+ cells 1 week after transplantation, suggesting the dedifferentiation of the labeled adipocytes (31). The results suggest that human fat cells can undergo dedifferentiated in physiological conditions. Additional studies have found that fat cells near breast cancer cells undergo phenotypic transformation from fat cells to fibroblast-like cells, and elongated cells with fat droplets have been observed in clinical cases of breast cancer. Further characterization of these cells shows that they are highly expressing FSP-1 but not α-SMA (6). This differs from the development of fibroblast-like cell phenotypes of ASCs, which is associated with α-SMA overexpression activated by surrounding breast cancer cells (32, 33). This result also further supports the notion that dedifferentiation of adipose cells occurs in human adipose tissue.

### Apparent morphological changes

During ceiling culture, adipocytes gradually lose a significant amount of lipids, with their nuclei becoming more concentrated and their shapes becoming more elongated (4, 5, 34). The electron microscopic characteristics of DFAT cells are very similar to those of stromal vascular fraction (SVF)-derived mesenchymal stem cells, which exhibit developed Golgi complexes, short chains of rough endoplasmic reticulum, small LDs, small mitochondria, lysosomes, and small glycogen particles. The nuclei are fusiform and have smooth edges (4).

**Fig. 1:**
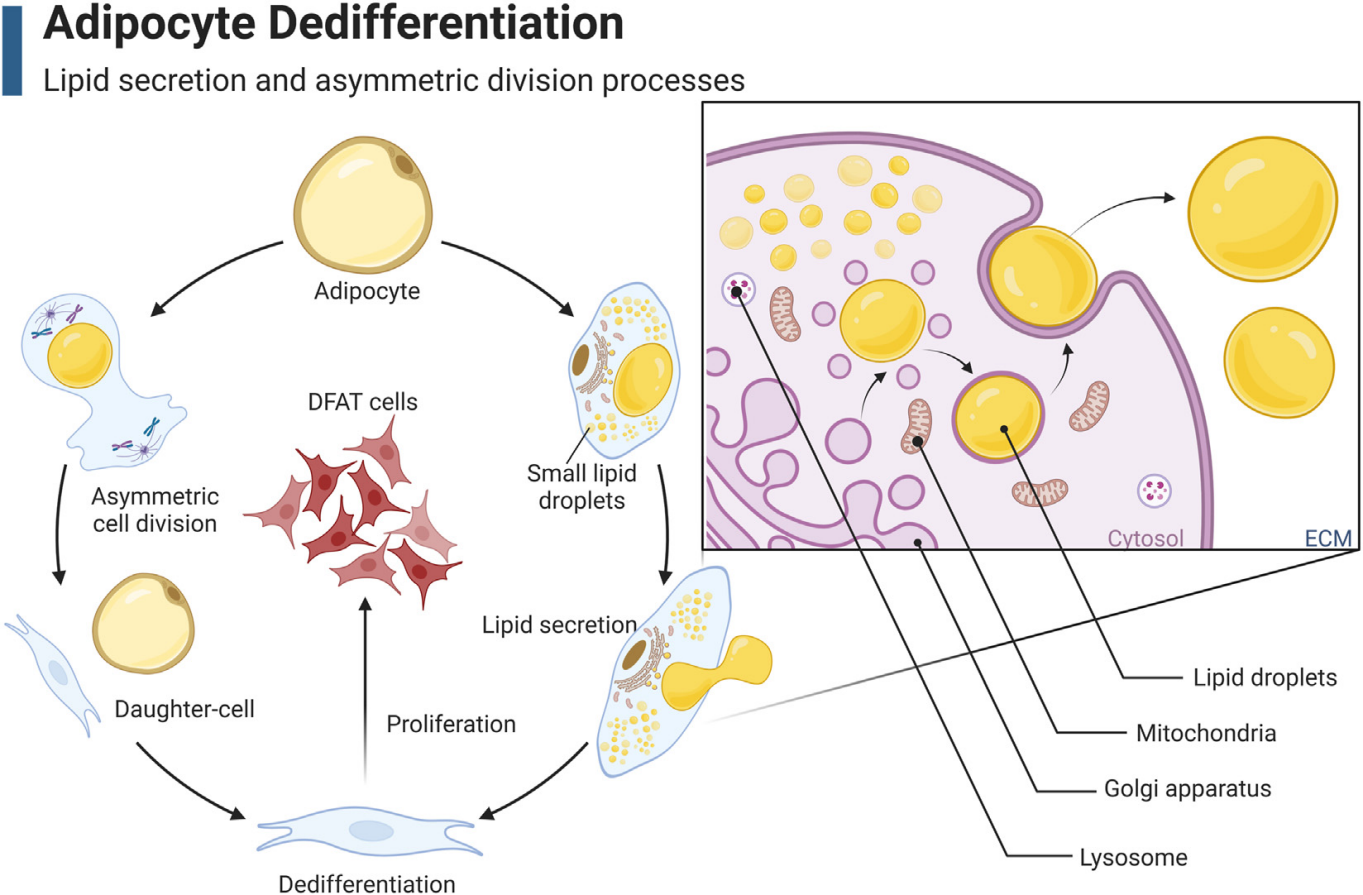
Lipid secretion and asymmetric division processes during adipocyte dedifferentiation. Mature adipocytes expel lipid droplets through a dual mechanism involving lipid secretion and asymmetric division. During lipid secretion, the rough endoplasmic reticulum, Golgi apparatus, mitochondria, and lysosomes are integral to the secretion process. The asymmetric division of adipocytes results in the generation of a single adipocyte alongside a lipid droplet-devoid daughter cell, which undergoes subsequent dedifferentiation to give rise to DFAT cells endowed with proliferative capacity. DFAT, dedifferentiated fat.

Previous studies have shown that adipocyte dedifferentiation is not caused by a gradual loss of lipids (e.g., lipidolysis) but a phenomenon called “lipid secretion” (5). In the early stages of adipocyte dedifferentiation, a distinctive three-layer plasma membrane gradually forms around most LD vacuoles (5). At this time, a group of developed organelles appeared in the cytoplasm, including secretory mitochondria, accumulated rough endoplasmic reticulum, and a hypertrophic Golgi complex, indirectly supporting the hypothesis of lipid secretion (Fig. 1). Interestingly, this “lipid secretion” phenomenon was observed in both subcutaneous adipose tissue explanted in matrix glue and obese adipose tissue, suggesting that adipocytes dedifferentiate in both fat transplants and obese tissue (5). Recent research has developed a microscopically mounted ceiling culture chip that enables long-term dynamic observation of fat cells while maintaining stable ceiling culture conditions (35). Studies have shown that during adipocyte dedifferentiation, dynamic cell deformation is accompanied by LD secretion. Moreover, this LD secretion is accompanied by dynamic deformation of the LD. Initially, the LD appears flat and trembles under mechanical stress; when it is squeezed out of the cell, it returns to its spherical shape (35). More importantly, the LD secretion process, characterized by oscillation and extrusion, may be mediated by intracellular actin. In the prosecretory phase, the cells form a pseudopod at the top, and actin filaments are distributed inside the LD, forming an actin shell. Occasionally, actin shells develop pores of varying sizes along the LD surface. When the secretion phase begins, these actin-enclosed pores merge into one or two larger pores. These larger pores serve as the openings through which the LD is extruded during the secretory phase. During secretion, actin contraction pushes the plasma membrane-wrapped LD out, leaving behind a hollow actin ring structure. These voids, resulting from LD secretion, are a unique feature of dedifferentiated fat cells and are not present in normal fibroblasts (35).

Additionally, during the process of ceiling culture, Matsumoto *et al.* found that some fibroblast-like cells were generated from mature adipocytes through asymmetric division by performing BrdU detection and time-lapse fluorescence microscopy (36). This division process resulted in the creation of one lipid-filled adipocyte and one lipid-free subcell. Subsequently, these lipid-free cells dedifferentiated, eventually giving rise to DFAT cells (36). This asymmetric division phenomenon has also been observed during the dedifferentiation of mature adipocytes in cattle and pigs (37). Hence, the dedifferentiation of mature adipocytes appears to result from two distinct phenomena: lipid secretion and asymmetric division (Fig. 1).

### Genetic and phenotypic changes

DFAT cells undergo significant changes in gene expression during their transition to a fibroblast-like phenotype. These gene changes can be classified into several categories. The first category is the downregulation of lipid metabolism-related genes. Genes related to lipid metabolism are significantly adjusted during the dedifferentiation process. This adjustment includes the downregulation of genes associated with adipogenic differentiation, lipid synthesis, and fatty acid degradation, as well as the up-regulation of genes involved in lipid breakdown (38, 39, 40). Secondly, expression of stem cell markers is upregulated in DFAT cells. This includes the upregulation of genes associated with embryonic stem cells and reprogramming genes, which occurs together with restoration of a hypomethylation status similar to that of stem cells (5, 41, 42). Thirdly, genes related to the cell cycle and proliferations are upregulated in DFAT cells. These upregulated genes are associated with processes such as mitosis, M phase, cell division and cell cycle progression (38). In vitro experiments have shown that DFAT cells exhibit similar proliferation abilities as ASCs (41). Fourthly, genes related to lineage differentiation are upregulated. These upregulated genes encompass all three germ layers, indicating that DFAT cells can differentiate into various cell lineages (38, 43). The last category is the upregulation of genes related to cellular processes. Genes involved in processes such as cell motility, cell migration, tissue development, cell growth, cell proliferation, cell morphogenesis and shape changes are upregulated during adipocyte dedifferentiation (38). These gene changes help explain why DFAT cells exhibit the similarities in proliferative capacity, morphological features and functional characteristics as ASCs (Table 1).

**Table 1:** Changes of gene expression in DFAT cells

Gene Types	Representative genes	Expression	Reference
Adipogenic differentiation	*PPARγ*, *C/EBPβ*, *C/EBPα*	Downregulation	(38, 39, 40)
Lipolysis	*LEP*, *LIPE*, *LPL*, *CES1*, *LIPA*, *PNPLA2*, *MGLL*	Downregulation	(38, 39, 40)
Lipid synthesis	*DGAT2*, *CIDEA*, *LPIN1*, *CHPT1*, *ACSS2*, *ACSL1*, *ANGPTL4*, *AGPAT1*, *AGPAT6*, *ABHD5*, *CDS1*, *SLC2A4*	Downregulation	(38, 39, 40)
Fatty acid degradation	*ADIPOQ*, *PPARα*, *PDK4*, *FABP4*, *FABP3*, *ACAA1*, *PHYH*, *ACSL1*, *PCCA*	Downregulation	(38, 39, 40)
Fatty acid synthesis	*FASN*, *ACSL1*	Downregulation	(38, 44)
Lipid transport	*RBP4*, *RBP1*, *APOE*, *CERT1*	Downregulation	(38, 44)
Embryonic stem cell marker	*NANOG*, *TBX1*, *SOX17*, *GATA4*	Upregulation	(5, 41, 42, 43)
Reprogramming	*OCT4*, *C-MYC*, *KLF4*, *SOX2*	Upregulation	(5, 41, 42, 43)
Regulation of differentiation	*SFRP2*, *HEY2*, *DLX2*, *AEBP1*, *PEG10*, *RUNX1*, *FZD7*, *IGFBP5,ID4*, *ID2*, *RUNX2*, *SOX9*	Upregulation	(38, 43)
Cell proliferation	*SERPINE1*, *VEGFC*, *CDH2*, *MDK*, *TIMP1*, *IL-18*, *IL-33*, *PLAU*, *ARNT2*, *TNFRSF12A*	Upregulation	(38)
Cell movement and migration	*SFRP2*, *CNN1*, *FHL2*, *CDH11*, *SERPINE1*, *SERPINE2*, *TNC*, *INHBA*	Upregulation	(38)
Cell morphogenesis and shape change	*BASP1*, *CDH11*, *SERPINE1*, *TNC*, *INHBA*, *ITGA8*, *VEGFC*, *SPP1*, *ITGA5*	Upregulation	(38)

*ABHD5*, *Abhydrolase domain containing 5*; *ACAA1*, *acetyl-CoAacyltransferase 1*; *ACSS2*, *acyl-CoAsynthetase short chain family member 2*; *ACSL1*, *acyl-CoAsynthetase long chain family member 1*; *ADIPOQ, Adiponectin*; *AEBP1*, *AE binding protein 1*; *AGPAT(1,6)*, *1-acylglycerol-3-phosphateO-acyltransferase (1,6)*; *ANGPTL4*, *angiopoietin like 4*; *APOE*, *apolipoprotein E*; *ARNT2*, *aryl hydrocarbon receptor nuclear translocator 2*; *BASP1*, *brain abundant membrane attached signal protein 1*; *CCAAT/enhancer binding protein(α/β)*; *CDH(2,11)*, *Cadherin(2,11)*; *CDS1*, *CDP-diacylglycerolsynthase 1*; *CERT1*, *ceramide transporter 1*; *CES1*, *carboxylesterase 1*; *CHPT1*, *choline phosphotransferase 1*; *CIDEA*, *cell death inducing DFFA like effector A*; *C-MYC*, *MYCproto-oncogene*; *CNN1*, *Calponin 1*; *DGAT2*, *diacylglycerol O-acyltransferase 2*; *DLX2*, *distal-lesshomeobox 2*; *FABP(3,4)*, *fatty acid binding protein(3,4)*; *FASN*, *fatty acid synthase*; *FZD7*, *frizzled class receptor 7*; *FHL2*, *four and a half LIM domains 2*; *GATA4*, *GATA binding protein 4*; *HEY2*, *Hes related family BHLH transcription factor With YRPW motif 2*; *ID(2,4)*, *inhibitor of DNA binding(2,4)*; *IGFBP5*, *insulin like growth factor binding protein 5*; *INHBA*; *inhibin subunit beta A*; *ITGA5*, *integrin subunit alpha 5*; *ITGA8*, *integrin subunit alpha 8*; *KLF4*, *KLF transcription factor 4*; *LIPA*, *lipase A*; *LEP*, *leptin*, *LIPE*, *lipase E*; *LPL*, *lipoprotein lipase*; *LPIN1*, *Lipin 1*; *MGLL*, *monoglyceride lipase*; *MDK, Midkine*; *NANOG*, *Nanog homeobox*; *OCT4*, *octamer-bindingprotein 4*; *PCCA*, *propionyl-CoAcarboxylase subunit alpha*; *PDK4*, *pyruvate dehydrogenase kinase 4*; *PEG10*, *paternally expressed 10*; *PHYH,phytanoyl-CoA2-hydroxylase*; *PPAR(α,γ)*, *peroxisomeproliferator-activatedreceptor γ*; *C/EBP(α,β)*, *PNPLA2*, *patatin like phospholipase domain containing 2*; *RUNX(1,2)*; *PLAU*, *plasminogen activator*; *RBP(1,4)*: *retinol binding protein(1,4)*; *RUNX family transcription factor(1,2)*; *SERPINE(1,2)*, *Serpin family B member(1,2)*; *SERPINE1*, *Serpin family E member 1*; *SFRP2*, *secreted frizzled related protein 2*; *SOX(2,9,17)*, *SRY-Boxtranscription factor (2,9,17)*; *SLC2A4*, *solute carrier family 2 member 4*; *SPP1*, *secreted phosphoprotein 1*; *TBX1*, *T-box transcription factor 1*; *TIMP1*, *tissue inhibitor of metalloproteinases 1*; *TNC*, *Tenascin C*; *TNFRSF12A*, *TNF receptor superfamily member 12A*; *VEGFC*, *vascular endothelial growth factor C*.

During the dedifferentiation of mature adipocytes, the cells shed large granular LDs and transformed into fibroblast-like forms. Their lipid metabolism and secretion capacities differed significantly from those of mature adipocytes. Multiple experiments have observed the downregulation of key regulators of adipogenesis and adipodifferentiation, such as peroxisome proliferator-activated receptor gamma *(PPARγ)* and CCAAT/enhancer binding protein alpha *(C/EBPα)*, indicating a return to predifferentiation gene expression (36, 38, 39, 40). Under specific induction conditions, *PPARγ* and *C/EBPα* can be reaccumulated, allowing the cells to redifferentiate into adipocytes (45, 46). In terms of fatty acid oxidation, dedifferentiation of adipocytes has been observed in a mouse model with Ad/N1ICD. Lipidomics analysis revealed the downregulation of fatty acid oxidation, lipid uptake, and oxidation pathways in Ad/N1ICD adipose tissue (28). Transcriptomic analysis further demonstrated the downregulation of *PPARα* and downstream peroxisome-associated genes, such as acetyl-CoA acyltransferase, phytanoyl-CoA 2-hydroxylase, and acyl-CoA synthase long-chain family member 1 (38) Regarding lipid synthesis and decomposition, studies have found that Epstein-Barr virus-infected adipocytes undergo dedifferentiation accompanied by increased mRNA expression of adipose triglyceride lipase (ATGL), hormone-sensitive lipase, and decreased fatty acid synthase expression. Western blot analysis showed the downregulation of AMPK, mTOR, and p70S6K1, while ATGL, hormone-sensitive lipase, and monoacylglycerol lipase were upregulated (47). The downregulation of the lipid synthesis pathway was also noted in Ad/N1ICD adipose tissue ^.28^ Additionally, melanoma cell-cocultured adipocytes showed decreased expression of fatty acid desaturase, sterol-C4-methyl oxidase-like, and fatty acid synthase during dedifferentiation (47). Compared to mature adipocyte, these studies indicated a decrease in lipid synthesis capacity and an increase in lipid decomposition capacity in dedifferentiated adipocytes. In terms of lipid secretion function, recent studies have shown that paracancer adipocytes can transform into cancer-associated adipocytes after interacting with breast cancer cells, exhibiting a dedifferentiated phenotype with increased expression of tumor-promoting adipocytokines such as leptin and resistin, and significantly decreased expression of the antitumor adiponectin (48). However, adipocytes cocultured with melanoma cells exhibited decreased expression of adipokines, suggesting that the lipid secretion of dedifferentiated adipocytes may fluctuate based on environmental factors (47). Nevertheless, dedifferentiation of adipocytes in various environments is consistently accompanied by an increase in free fatty acids and lipid breakdown products, such as glycerol (28, 47, 48, 49).

The most recent study examined pork-derived mammary adipose cells from five developmental stages (day 90 of gestation, G90; day 0 after lactation, L0; day 20 after lactation, L20; 2 days post natural involution, PI2; 7 days post natural involution, PI7) using single-nucleus RNA-seq and pseudotime analysis (50). During the transition from G90 to lactation (L0 and L20), most adipocytes gradually dedifferentiated from a highly differentiated state and remained dedifferentiated during lactation, which is consistent with previous observations in the mammary glands of pregnant mice (22, 51). The genes that is highly expressed during lactation mainly regulate cell morphogenesis and are significantly enriched in the classical insulin receptor signaling pathway and AMPK signaling pathway. In the two pathways, three key genes were highlighted: *Zfp36l1*, *Foxo1*, and *Lpin1* (50). Previous studies have confirmed that *Zfp36l1* inhibits intracellular fat synthesis (52, 53), *Foxo1* inhibits adipocyte differentiation (54), and *Lpin1* is more highly expressed in preadipocytes than in mature adipocytes (55, 56). Pseudotime analysis showed that during lactation (L0 and L20), there was a decreased expression of genes related to lipid synthesis, including regulation of lipid storage (e.g., *Cd36*), response to fatty acids (e.g., *Lpl*), and triglyceride metabolism (*Abca1*), compared to G90 and involution (PI2 and PI7) (50).

Inflammation and insulin resistance caused by chronic hypoxia are important characteristics of obese adipose tissue. Previous studies have shown that cytokines such as TNF-α (17) and monocyte chemoattractant protein 1 (MCP-1) (57), which are elevated in an obese environment, can reduce the expression of adipogenic genes in vitro and induce dedifferentiation of adipose cells. Other studies have also observed the phenomenon of lipid secretion in obese adipose tissue (5), suggesting the possibility of adipocyte dedifferentiation in obese tissue. In recent single-nucleus RNA-seq on the epididymal white adipose tissue of obese and lean mice, three adipocyte subsets were defined: (1) lipogenic adipocytes with high expression of genes related to de novo lipogenesis (such as *Acaca* and *Acly*); (2) lipid-scavenging adipocytes (LSAs) with high expression of genes related to fat absorption and transport (e.g., *Cd36* and *Apoe*); (3) stressed LSAs (SLSAs) expressing genes related to hypoxia and autophagy (e.g., *Hif1a* and *Rab7*). Interestingly, in high-fat diet induced obesity, the relative proportion of LSA and SLSA subgroups increased, while the relative proportion of lipogenic adipocytes decreased. Moreover, lipidomics has shown that the genes suppressed in the LSA and SLSA subpopulations during obesity include those related to mature adipocyte functions, such as lipid biosynthesis (e.g., *Lpl* and *Plin1*), insulin response (e.g., *Insr* and *Irs1*), and adipocyte differentiation (e.g., *Pparg* and *Nr3c1*) (58). These results suggest a transition from mature adipocytes to adipocyte subtypes with stronger abilities for lipid secretion and transport and weaker abilities for lipid synthesis during dedifferentiation.

Current studies generally observe the lipid quantity and droplet structure of adipocytes during dedifferentiation using Oil Red O staining or electron microscopy (5, 7). These experiments consistently find that mature adipocytes enhance the lipid secretion process during dedifferentiation and switch to continuous lipid-secretion until they finish dedifferentiation with almost no fat droplets. However, there are currently no studies directly differentiating DFAT cells from mature adipocytes, lipid-secreting adipocytes, or fibroblast adipocytes based on their intracellular lipid contents. Future studies could utilize histological techniques such as BODIPY fluorescence staining (59) and mass spectrometry like LC-MS (60) to distinguish DFAT cells from adipocytes with their different lipid types and contents. By defining time points in the dedifferentiation process, researchers can further elucidate the changes in lipid metabolism during this transition.

Several research groups have utilized flow cytometry to identify cell surface antigens of DFAT cells, ASCs, and bone marrow mesenchymal stem cells (BMSCs) (36, 41, 61, 62, 63, 64, 65, 66, 67). The results of numerous studies consistently demonstrate that all three cell types exhibit positivity for the surface antigens CD9, CD13, CD29, CD44, CD54, CD90, CD105, and CD166, as well as HLA-ABC, but are negative for CD14, CD66b, CD133, and HLA-DR (36, 41, 61, 62, 63, 64, 65, 66, 67). To better differentiate these cell types, a table summarizing the differences in cell surface markers among DFAT cells, ASCs, and BMSCs has been compiled (Table 2). However, it is essential to note that variations in species, cell sources, isolation techniques, and culture methodologies can lead to differences in the identification of the same cell surface markers across various experiments.

**Table 2:** Summary of the characteristics of dedifferentiated adipocytes (DFAT cells), adipose-derived stem cells (ASCs), and bone marrow mesenchymal stem cells (BMSCs)

Characteristics			DFAT Cells	ASCs	BMSCs	Rf
Shape			Fibroblastoid or long fusiform			(41, 42)
Origin			Mature adipocytes	Mesodermal cells	Mesodermal cells	(5, 41, 42)
Acquisition method			Adipose tissue collection; adipocyte isolation; ceiling culture	Adipose tissue collection; collagenase digestion; filtration and centrifugation; SVFs collection; cell culture	Bone marrow extraction; density gradient centrifugation; mononuclear cell layer collection; cell culture	(2, 41, 42)
Differentiation lineage	Similarities		All three cell types can differentiate into adipocytes, chondrocytes, osteoblasts, myocytes, vascular endothelial cells, hepatocyte and neurogenic-lineage cells.			(8, 9, 10, 36, 41, 42, 46, 68, 69)
	Differences		Better adipogenic osteogenic and neurogenic differentiation potential than ASCs	Similar or better chondrogenic differentiation potential than DFAT cells	-	(3, 36, 62, 70)
Cell surface makers	Similarities		All three cell types are positive for CD9, CD13, CD29, CD44, CD54, CD90, CD105, CD166, and HLA-ABC, but are negative for CD14, CD66b, CD133, and HLA-DR.			(36, 41, 61, 62, 63, 64, 65, 66, 67)
	Differences	–	CD11b,CD31,CD34,CD45,CD106,CD117,CD140b,α-SMA	CD11b,CD31,CD34,CD45,CD49d,CD106,CD117	CD11b,CD31,CD34,CD45,CD49d,CD106,CD117	DFAT cells: (36, 61, 62, 64, 67) ASCs: (36, 61, 62, 63, 64, 66, 67) BMSCs: (64, 65)
		±	CD140b	CD11b,CD31,CD34,CD45,CD49d,CD106,α-SMA	-	DFAT cells: (67) ASCs: (36, 61, 63, 66, 67)
		+	CD49d	CD49d,CD140b,α-SMA	CD31,CD106,CD117	DFAT cells: (36, 61, 67) ASCs: (36, 61, 67) BMSCs: (36, 65)

+: positive expression and –: negative expression. ± partial expression.

### Redifferentiation potential

Because DFAT cells exhibit gene transcription characteristics reminiscent of pluripotent stem cells, researchers have comprehensively examined their redifferentiation potential. Notably, DFAT cells can differentiate into various cell lineages under both in vivo and suitable in vitro culture conditions. These lineages include adipocytes, (41, 45, 46, 68, 70) chondrocytes, (41, 45, 71, 72, 73) osteoblasts, (10, 41, 70, 74, 75, 76, 77) myocytes, (9, 69, 78) vascular endothelial cells, (79, 80, 81) hepatocyte (5) and neurogenic-lineage cells. (41, 82, 83) Intriguingly, when evaluated by Oil Red O staining, alkaline phosphatase activity, and mineralization assays in vitro, DFAT cells exhibited a better adipogenic and osteoblastic differentiation potential than ASCs (62, 70, 84). Furthermore, as determined by Alcian blue analysis, DFAT cells and ASCs demonstrated a similar chondrogenic differentiation potential (84). These findings underscore the multifaceted differentiation potential of DFAT cells, which surpasses that of other mesenchymal stem cells. Consequently, DFAT cells represent a valuable source of donor cells for applications in tissue engineering.

## Potential Targets for Regulating Adipocyte Dedifferentiation

Since the initial revelation that mature adipocytes can dedifferentiate, (3) numerous researchers have studied adipocyte dedifferentiation. In this review, we provide a comprehensive summary of adipocyte dedifferentiation, encompassing observations made in various physiological (21, 22) and pathological (6, 7, 26, 85) contexts, as well as instances induced under controlled artificial conditions (14, 15, 20, 86). Furthermore, we delineate the specific signaling pathways responsible for inducing dedifferentiation in these distinct environments, with detailed information in Table 3.

**Table 3:** Adipose dedifferentiation and its specific signaling pathways in different environments

Treatment	Results	Signaling Pathway	Rf
Wnt3a	Wnt3a promotes adipocyte dedifferentiation in vitro	Wnt/β-catenin	(14)
WISP2	WISP2 activates Wnt pathway to promote adipocyte dedifferentiation in vitro	Wnt/β-catenin	(86)
Breast tumor cells	Wnt3a secreted by breast tumor cells transforms adipocytes into adipocyte-derived fibroblasts (ADF) in vitro and in vivo; ADF accelerates the progression of breast cancer	Wnt/β-catenin	(6)
Hyperosmotic stress or mechanical compression	Hyperosmotic stress or mechanical compression promotes adipocyte dedifferentiation in vitro; The compression-induced dedifferentiated adipocytes (CiDAs) accelerate the progression of breast cancer	Wnt/β-catenin	(20)
MiaPaCa2 cancer cells	MiaPaCa2 cancer cells secrete wnt5a to dedifferentiate adipocytes in vitro	WNT5a/ROR2/c-JUN/AP1	(7)
TGF-β1	TGF-β1 promotes adipocyte dedifferentiation in vitro	TGF-β/SMAD	(15, 26)
Bleomycin-treated mice to mimic scleroderma	TGF-β drove subcutaneous adipocytes to undergo fibrogenic differentiation preferentially in vivo	TGF-β	(26)
Adiponectin-Cre-N1ICD mice to activate Notch signaling	Activation of Notch signaling leads to adipocyte dedifferentiation and transformation into liposarcoma	Notch	(85)
Cold exposure	Cold-exposed adipocytes transformed into fibroblast-like cells and upregulated brown adipocyte transforming genes	-	(21)
FIZZ1	FIZZ1 (Found in inflammatory zone 1) causes dermal fibrosis by activating Notch pathway for the transdifferentiation of adipocytes into myofibroblasts	Notch	(18)
OSI-906 to block insulin signaling	Insulin negatively regulates dedifferentiation of mouse adipocytes in vitro	Insulin-PI3K-AKT-mTOCR1	(16)
Autophagy inducer	Autophagy promotes adipocyte dedifferentiation triggered by poor insulin signaling	Insulin-PI3K-AKT-mTOCR1-autophagy	(16)
TNF-α	TNF-α induced dedifferentiation of adipocytes	TNF-α-MAPK-PPARγ	(17)
AdipoChaser-LacZ model to trace dedifferentiation and redifferentiation	Adiponectin positive adipocytes can undergo dedifferentiation and redifferentiation in mammary gland	-	(22)

**Fig. 2:**
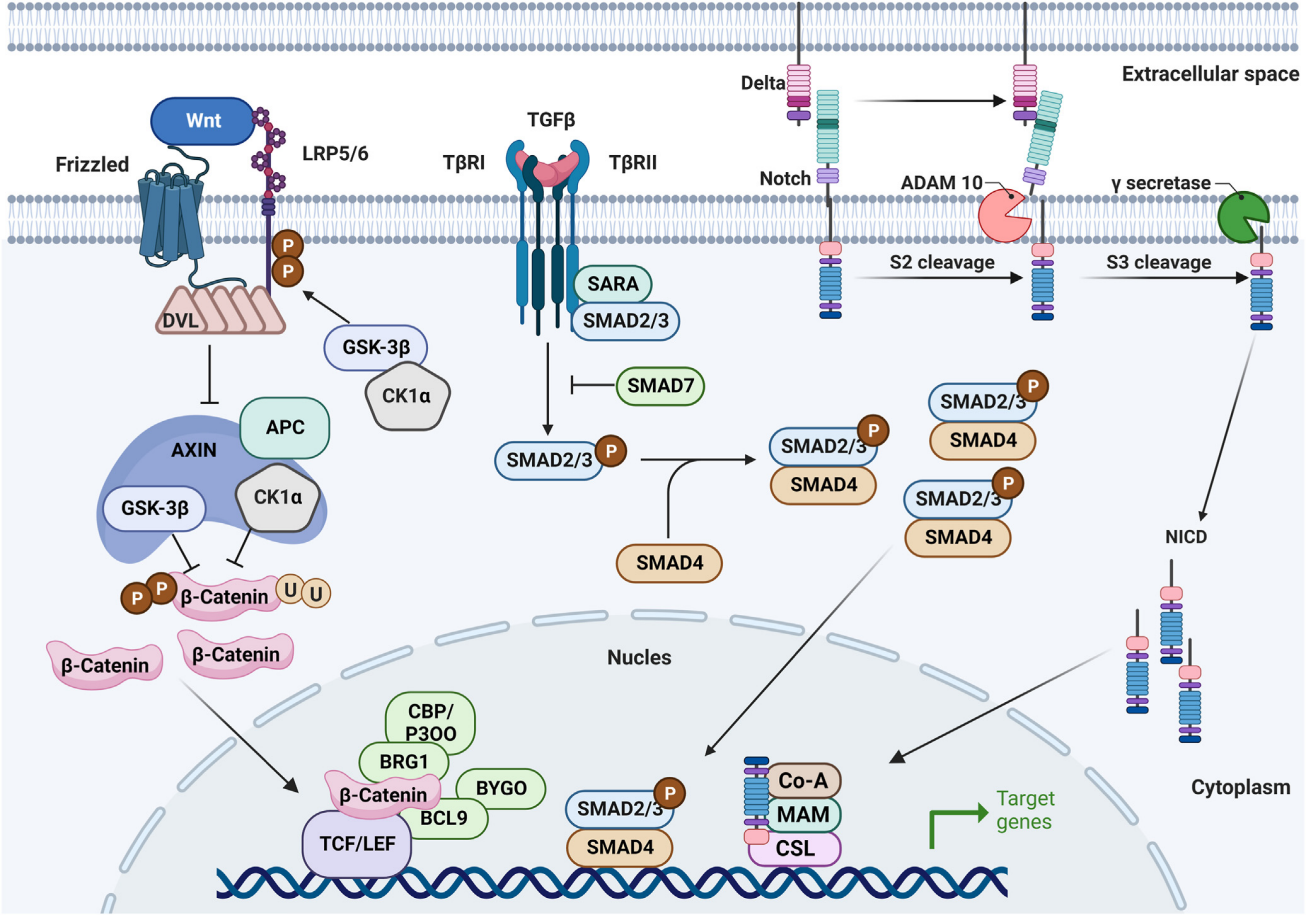
Schematic representation of key nodes in adipocyte dedifferentiation induced by the three signaling pathways.

The primary signaling pathways implicated in adipocyte dedifferentiation encompass the Wnt, TGF-β, and Notch pathways (Fig. 2). Activation of these pathways results in the downregulation of lipogenic gene expression in mature adipocytes, leading to their acquisition of a fibroblast-like morphology (5, 6, 38, 41, 63). We investigated pivotal components of these signaling pathways to identify prospective targets for promoting adipocyte dedifferentiation.

### β-catenin

β-catenin plays a crucial role in transmitting classical Wnt signals to the nucleus (87). In the absence of Wnt signaling, β-catenin in the cytoplasm undergoes phosphorylation and is marked by a protein complex containing axis inhibition protein (AXIN), adenomatous polyposis coli protein (APC), glycogen synthase kinase (GSK)-3, and casein kinase (CK)1. Phosphorylated β-catenin is subsequently degraded by the proteasome. However, activation of the Wnt/β-catenin signaling pathway triggers a cascade of downstream events. AXIN binds to phosphorylated lipoprotein receptor-associated protein, leading to disruption of the protein complex. Unphosphorylated β-catenin accumulates in the cytoplasm and subsequently migrates into the nucleus. In the nucleus, β-catenin forms complexes with T cell factor/lymphoid enhancer-binding factor (TCF/LEF) family transcription factors and Legless family dock proteins (*BCL9* and *BCL9L*), along with PYGO family coactivators (*PYGO1* and *PYGO2*). This complex induces the transcription of specific target genes that are inhibitors of *PPARγ* and *C/EBPα*, which are key transcription factors involved in lipogenesis. These target genes include cyclin D1 *(CCND1)*, *C-MYC*, *PPARδ*, and chicken ovalbumin upstream promoter transcription factor II (*COUP-TFII*). *C-MYC* prevents fat formation by inhibiting expression of *C/EBPα*. Additionally, increased expression of *CCND1* and *PPARδ* inhibits lipogenesis by inhibiting *PPARγ* activity. *COUP-TFII* forms a complex with the *SMRT* inhibitor in the first intron downstream of the first exon of *PPARγ1* and *PPARγ2* mRNA. This complex causes local nuclear histones to have low acetylation levels and thereby inhibits *PPARγ* gene expression (Fig. 2) (88, 89, 90).

Activation of the Wnt/β-catenin signaling pathway induces adipocyte dedifferentiation, as demonstrated in various experiments (14, 20). Gustafson *et al.* induced adipocyte dedifferentiation by introducing exogenous Wnt3a. During this process, Wnt3a reduced the degradation of free β-catenin, leading to the accumulation of β-catenin in the cytoplasm. This accumulated β-catenin translocated to the nucleus, where it induced the expression of target genes that inhibited the expression of *PPARγ*, a key regulator of adipocyte differentiation (14). Additionally, Li *et al.* successfully induced adipocyte dedifferentiation by simulating the physical tumor microenvironment through the application of high physical compression force and high osmotic pressure. During this process, they observed the nuclear translocation of β-catenin using immunostaining techniques (20). These findings suggest that enhancing the expression and dephosphorylation of β-catenin or inhibiting its degradation are novel strategies to regulate adipocyte dedifferentiation.

### SMAD family member 3 (SMAD3)

SMAD3 is a pivotal component of the TGF-β1 signaling pathway, which plays a fundamental role in regulating various cellular processes (20, 91, 92). The TGF-β1 signaling pathway is initiated when the TGF-β1 ligand binds to TGF-β-associated type II receptor (TβRII), leading to recruitment and activation of TGF-β-associated type I receptor (TβRI) by TβRII. TβRI subsequently phosphorylates downstream substrates, including SMAD2 and SMAD3. Once phosphorylated, SMAD2 and SMAD3 form complexes with SMAD4 and translocate from the cytoplasm to the nucleus. Inside the nucleus, the SMAD complex regulates the expression of specific genes by interacting with various transcription factors. In the context of adipocyte biology, SMAD3 acts on key transcription factors like *C/EBPβ* and *C/EBPδ*, leading to suppression of their transcriptional activity. This downregulation of *C/EBPβ* and *C/EBPδ* inhibits the expression of *PPARγ* mRNA, which is pivotal for adipocyte differentiation. (61–63) It is important to note that SMAD7, an inhibitory SMAD, plays a crucial role in regulating the TGF-β1/SMAD3 signaling pathway. SMAD7 interferes with SMAD3 activation by promoting the degradation of TβRI and preventing SMAD3 phosphorylation (Fig. 2) (20).

Recent studies have provided insights into the regulatory role of the TGF-β1/SMAD3 signaling pathway in the process of adipocyte dedifferentiation. When TGF-β1 acts on mature adipocytes, it effectively reduces the expression of lipogenic genes, prompting their transformation into a fibroblast-like state. Interestingly, the expression of SMAD3, a crucial component of the TGF-β1 signaling pathway, is upregulated during this dedifferentiation process (15, 26). This suggests that SMAD3 is intimately linked to dedifferentiation of adipocytes. In further support of this notion, studies involving KO of SMAD3 demonstrated that the stimulatory effect of TGF-β1 on dedifferentiation of adipocytes is notably reduced when SMAD3 is absent (93). Therefore, SMAD3 appears to be essential for TGF-β1-induced adipocyte dedifferentiation. Thus, strategies that enhance SMAD3 expression, promote the phosphorylation of SMAD3, or inhibit the action of SMAD7 (an inhibitory component of the pathway) may offer potential avenues for inducing adipocyte dedifferentiation. It is crucial to be aware that continuous activation of the TGF-β1/SMAD3 signaling pathway can lead to transdifferentiation of adipocytes into myofibroblasts. This transformation can contribute to the development of diseases characterized by intratissue fibrosis, such as idiopathic pulmonary fibrosis and scleroderma (26, 94). Therefore, when manipulating this pathway to induce adipocyte dedifferentiation, it is essential to consider the potential implications and risks, particularly in relation to fibrotic diseases (Fig. 2).

### Notch intracellular domain

Notch intracellular domain (NICD) is a crucial component of the Notch signaling pathway (44, 95). Notch signaling is typically activated when Notch receptors bind to Notch ligands. This binding leads to the cleavage of the Notch receptor, resulting in the release of NICD. NICD then translocates to the nucleus, where it binds to the DNA-binding protein RBPJ (recombination signal binding protein for immunoglobulin kappa J region). This interaction leads to the activation of various downstream target molecules, including hairy enhancer-of-split homolog-1 (*HES-1*), hairy/enhancer-of-split related with YRPW motif protein 1 (*HEY-1*), and pro adipocytokine 1 (*PREF-1*). Activation of *PREF-1*, particularly via the MEK/ERK signaling pathway, activates sex-determining region *SOX9*. *SOX9*, in turn, binds to the promoter regions of *C/EBPβ* and *C/EBPδ*, inhibiting promoter activity and preventing differentiation of adipocytes (95, 96, 97). Additionally, hairy enhancer-of-split homolog-1 inhibits the expression of *PPARγ* by targeting the E-box elements in *PPARγ* promoters (Fig. 2) (95, 96, 98).

The Notch signaling pathway is a key regulator of adipocyte dedifferentiation (28, 85, 99). Chartoumpekis *et al.* established a mouse model with adipocyte-specific overexpression of NICD (AD/NICD) and thereby enhanced Notch1 signaling. Their research revealed a gradual loss of fat droplets and emergence of an undifferentiated phenotype in mature adipocytes of AD/NICD mice (85). These findings suggest that promoting NICD expression or facilitating the hydrolytic release of NICD facilitates adipocyte dedifferentiation. However, it is crucial to note that activation of the Notch signaling pathway can trigger the conversion of adipocytes into liposarcoma (LPS) and subsequently promote the transformation of LPS into dedifferentiated LPS, which is characterized by heightened aggressiveness, metastatic potential, and lethality (28, 99). Consequently, further investigation is required to assess the safety of inducing adipocyte dedifferentiation via the Notch signaling pathway.

## Future and Outlook

### Purity and safety of DFAT cells

The higher purity of DFAT cells compared with ASCs may improve predictability in their applications, ultimately leading to improved safety and efficacy when they are used in cell therapy. ASCs are obtained through the expansion of a small number of stem cells from SVF, which comprises various cell types, including erythrocytes, macrophages, monocytes, endothelial cells, fibroblasts, pericytes, and mesenchymal cells (68, 100). Matsumoto *et al.* reported that human ASCs at passage 1 comprise 18.6% α-SMA-positive cells, 12.8% CD45-positive cells, 13.3% CD11b-positive cells, and 2.7% CD31-positive cells, indicating contamination by smooth muscle cells, lymphocytes, monocytes, and endothelial cells, respectively (24). In contrast, DFAT cells represent a relatively homogeneous cell population, with minimal contamination of SVF cells (0%–0.07%) (36).

However, it is important to note that improving cell purity remains a significant challenge. Currently, the commonly used methods to obtain DFAT cells are still the collagenase decomposition method and ceiling culture after isolation (101, 102, 103). However, during the separation of mature adipocytes, a small number of other cell types with undifferentiated morphology but similar buoyancy (such as preadipocytes, fibroblasts, and stem cells) may be coseparated and attached with mature adipocytes, resulting in the contamination of DFAT cell populations by other cell types (104, 105, 106). The purity of mature fat cells isolated from adipose tissue can be a key parameter for stable growth and passage.

**Fig. 3:**
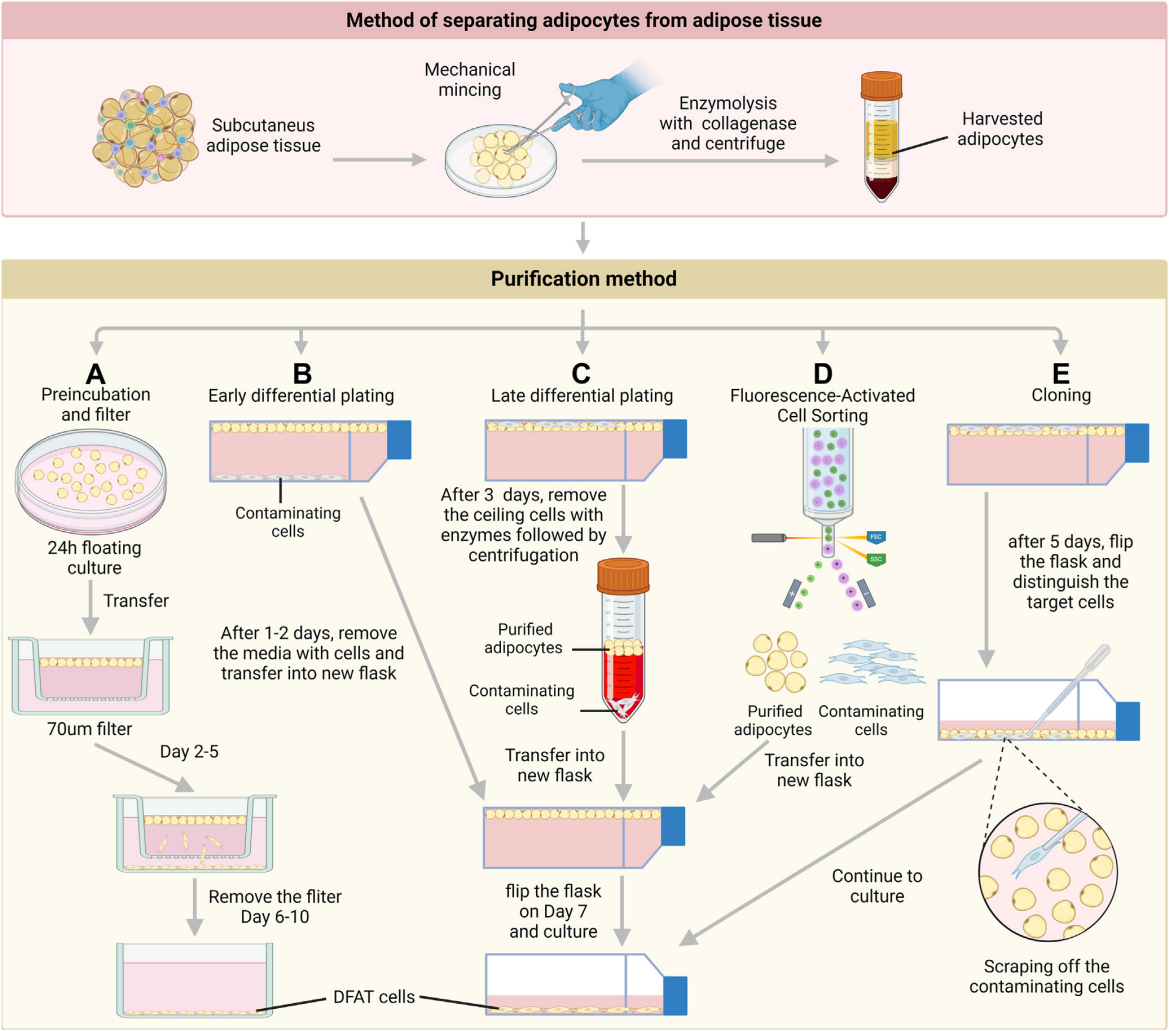
Method for increasing the purity of isolated mature adipocytes. A: Preincubation and filter. B: Early differential plating. C: Late differential plating. D: Fluorescence-activated cell sorting. E: Cloning.

Several key points could help improve DFAT cell purity. Firstly, the use of collagenase to separate fat cells requires thorough digestion. Studies have explored the appropriate collagenase concentration and enzymolysis time to obtain more SVF cells (107). However, whether the adjustment of collagenase concentrations and enzymolysis time can obtain higher purity needs further experimental exploration. Secondly, in the process of ceiling culture, mature adipocytes can be further purified by early differential plating (Fig. 3B), late differential plating (Fig. 3C), and cloning technology (Fig. 3E) (67, 108). After 1–2 days of ceiling culture, mature fat cells remained mostly suspended in the medium while cells with no lipid were attached to the bottom of the culture bottle. At this time, the suspended mature fat cells are transferred to a new culture bottle by early differential plating, leaving the contaminated cells attached to the bottom. On the third and fourth day, mature fat cells can adhere to the top surface but still preserve intracellular LDs. At this stage, digestion with trypsin and further centrifugation can be used to eliminate the contaminated cells. After being cultured for five days, the lipid-free cells can be labeled with a pipette under a microscope and scraped from the flask to purify the cell population (67, 108).

Another method of preparing DFAT cells has been reported to utilize preincubation and filters (Fig. 3A), in which isolated fat cells are incubated in a medium for 24 h to allow the underlayer cells shed and sink to the bottom. The top fat cells are then transferred to a medium in a six-well plate with a 70 μm filter and incubated for 5 days. DFAT cells pass through the filter and attach to the bottom of a Petri dish. This culture method not only improves the purity of DFAT cells but also significantly increased the early expression of pluripotency markers (43).

In addition, specific cell sorting techniques are necessary to address the cell purity problem in DFAT cell production (Fig. 3D). Fluorescent-activated cell sorting and magnetic-activated cell sorting are two of the most powerful techniques for separating desired cell types from mixed populations (109, 110, 111). Recent studies have described detailed fluorescent-activated cell sorting methods for the analysis and sorting of freshly isolated primary white, beige, and brown fat cells from human and mouse samples (112). Magnetic-activated cell sorting has been routinely used to isolate many cells types, including somatic stem cells (113), and has potential for adipocytes and DFAT separation. The comprehensive application of these different purification methods may help to screen out contamination cells groups and ensure the production of downstream DFAT cells for future clinical use.

The extensive morphological, functional, and molecular changes that occur during the dedifferentiation of DFAT cells (36, 41, 104) naturally raise concerns about whether these transformations might render the cells more susceptible to malignant transformation. Research conducted by Poloni *et al.* has provided valuable insights into this issue. Despite their high proliferation capacity, DFAT cells exhibited stable telomere lengths in comparison with mature adipocytes. Moreover, there was no transcription of human telomerase reverse transcriptase and consequently no telomerase activity, suggesting that both cellular transformation and senescence are avoided (114). The consistent expression levels of c-myc and p53, the inability to grow in an anchorage-independent manner, and the absence of DNA damage collectively suggest that these cells are safe (114). Furthermore, both in vitro and in vivo data indicate that DFAT cells do not form teratomas during proliferation (20, 43). These findings provide robust evidence supporting the safety profile of DFAT cells, alleviating concerns about their potential to undergo malignant transformation and form teratomas.

### Tackling obesity

Obesity is a global health issue affecting nearly 2 billion people and is associated with a significantly increased risk of developing various chronic diseases, including type 2 diabetes. The core problem of obesity is a set of metabolic syndromes, such as insulin resistance, inflammation, and fibrosis, which result from the pathological expansion of adipose tissue (115, 116). The pathogenesis of obesity has two prominent features. The first is hypoxia. Overexpansion of adipose tissue leads to decreased vascular density and an inadequate blood supply to the tissue. This causes cells to be in a chronic state of hypoxia, triggering immune and inflammatory responses. The second is inflammation. The hypoxic state in adipose tissue contributes to chronic inflammation, which further exacerbates the metabolic disturbances associated with obesity (117).

There were hypothesis that obesity led to a pathological increase in lipid accumulation in adipose tissue, pushing adipose cells away from the vascular system and triggering an inflammatory response. The development of Hypoxia is the basis for the initiation and progression of inflammation in obese adipose tissue (118, 119). Related experiments have directly detected low pO2 levels in adipose tissue in obese animal models and subjects. Adipose tissue of genetically obese (ob/ob and KKAy) mice and high-fat diet-induced obese mice was indirectly evaluated using a hypoxic probe system, revealing hypoxic regions margins (120, 121, 122). Immunostaining showed that macrophages recruited in obese patients were located in hypoxic regions, suggesting a close connection between hypoxia and inflammation (121). Additionally, pO2 levels in adipose tissue were measured using a needle-like optical fiber O2 sensor. The result indicated that pO2 levels in obese mice were significantly lower than in lean mice (121, 122). Tissue pO2 decline was also measured in obese subjects (123, 124). Furthermore, hypoxia-inducible factor 1α, a subunit of a key transcription factor involved in the cellular response to hypoxia (125), was significantly increased in obese adipose tissue, supporting the phenomenon of relative hypoxia in tissues (126).

Although there is no clear evidence that hypoxia induces adipocyte dedifferentiation, a study found that the expression of *ADIPOQ*, *FABP3*, *FABP5*, and *PPARGC1A* genes, which are highly correlated with fat cell function, was significantly downregulated in an RNA-Seq analysis of adipocytes treated with hypoxia (127). Additionally, pathways related to lipid metabolic function, involving glucose absorption, lipid oxidation and lipolysis, are significantly altered, suggesting that adipocytes may tend to dedifferentiate in hypoxic environments (127). Adipocyte dedifferentiation involves the transformation of adipocytes starts from a mature, lipid-filled state to a fibroblast-like predifferentiation state. During this process, fat cells lose significant lipid storage, reducing microenvironmental oxygen consumption and thereby improving hypoxia within the tissue. Therefore, inducing adipocyte dedifferentiation may be a new strategy to alleviate internal hypoxia in adipose tissue.

Recent studies have indicated that adipocyte dedifferentiation may mitigate insulin resistance and inflammation in adipose tissues through their interactions with macrophages. In normal adipose tissue, monocytes reside with adipocytes via delivery through the vascular system and are termed adipose tissue-resident macrophages (ATMs) (128). ATMs can be categorized into two distinct subgroups based on their unique cytokine profiles and cellular functions: the classically activated M1 subgroup and the alternatively activated M2 subgroup. During the onset and progression of obesity and related metabolic disorders, macrophages and adipocytes engage in cross-talk to regulate immune-metabolic homeostasis or disruption (129). Obesity can induce a phenotypic shift of ATMs from M2 to M1, with pro-inflammatory cytokines such as TNF-α, interleukin (IL)-1, and IL-6, which are particularly secreted by M1 ATMs, exacerbating insulin resistance and contributing to local or systemic metabolic dysfunction (129, 130, 131, 132). Extracellular vesicles, which are responsible for vital intercellular signaling mechanisms, play a pivotal role in this process (130). By contrast, IL-10, which is produced by M2 ATMs, alleviates TNF-α-induced insulin resistance (133).

Wang *et al.* recently reported that adipocytes in adipose tissue grafts could dedifferentiate and redifferentiate, resulting in reduced release of the pro-inflammatory cytokine IL-6 and promotion of M2 macrophage polarization. This phenomenon inhibits macrophage-mediated inflammation and insulin resistance (134). These findings provide a promising theoretical foundation for therapeutic approaches involving adipocyte dedifferentiation. Nevertheless, while it is evident that adipocyte dedifferentiation can induce phenotypic changes in ATMs, further investigation is required to elucidate the pathways leading to M2 macrophage polarization and the alterations that occur in adipocytes and ATM-secreted exosomes following dedifferentiation.

### Possible applications in fat transplantation

**Fig. 4:**
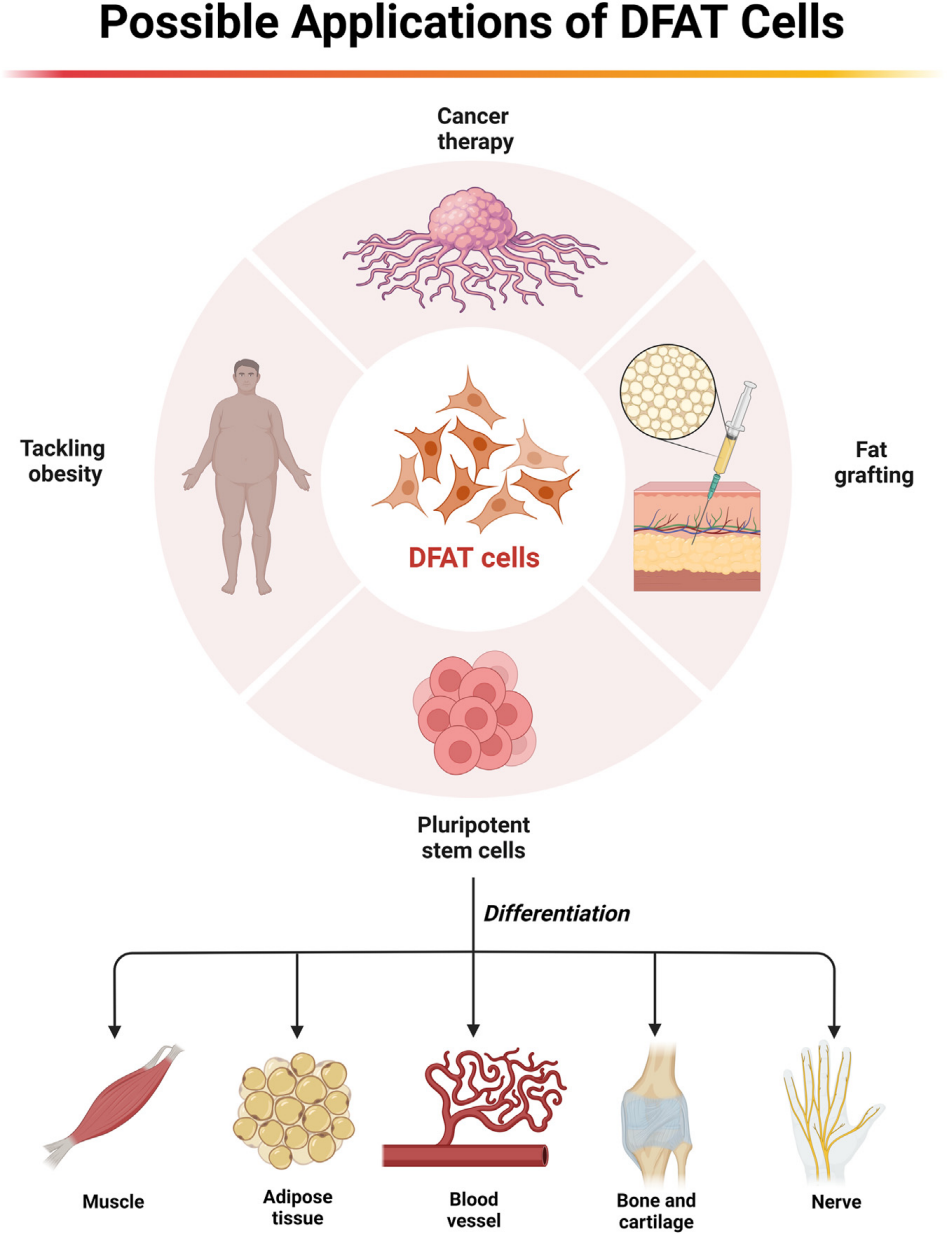
The regulation of adipocyte dedifferentiation holds promise for benefiting tumor therapy, obesity treatment, and enhancing the retention rate of transplanted fat. Furthermore, as a pluripotent stem cell source, dedifferentiated fat (DFAT) cells exhibit potential utility in the realm of regenerative medicine.

Autologous fat grafting is a widely employed clinical procedure (135, 136). Nonetheless, the predictability of fat retention following autologous fat grafting remains uncertain, (137) and the mechanisms governing the survival of transplanted adipocytes have not been fully elucidated. Current research predominantly proposes that adipocytes at the graft site may dedifferentiate after fat grafting. This dedifferentiated state is believed to confer enhanced resilience to the ischemic and hypoxic conditions encountered post-transplantation, favoring cell survival. Notably, recent studies also reported that adipocytes dedifferentiate following transplantation (31, 138, 139). Ma *et al.* successfully induced the dedifferentiation of a substantial number of mature adipocytes through mechanical force application in vivo (140). Subsequent retransplantation of tissue containing these dedifferentiated adipocytes markedly improved tissue retention compared with the control groups (140). Further investigations demonstrated that the injection of exogenous DFAT cells can stimulate angiogenesis at the graft site (140). In summary, there is evidence that promoting dedifferentiation of grafted fat enhances tissue retention post-transplantation. These findings hold promise for offering novel insights and methodologies for the clinical application of autologous fat grafting. However, it is important to note that Chartoumpekis *et al.* induced adipocyte dedifferentiation by specifically activating Notch signaling in the AD/NICD mouse model, resulting in lipodystrophy and nearly complete eradication of white adipose tissue (28, 85). The difference in adipose tissue retention may be associated with the proportion of dedifferentiated adipocytes in the tissue and the duration of induction. Thus, the dedifferentiation of adipocytes in adipose tissue presents a double-edged sword with regard to the survival of transplanted fat (Fig. 4).

### Potential targets for cancer therapy

In recent years, there has been growing awareness of dedifferentiation of tumor-associated adipocytes in the tumor microenvironments of various cancers, including breast cancer, (6) pancreatic cancer, (7) nasopharyngeal carcinoma, (49) and LPS (85). The challenging conditions within the tumor microenvironment, encompassing factors like hypoxia, inflammation, infection, and diverse forms of physical stress, robustly stimulate the reprogramming of precancerous adipocytes. Regrettably, this dedifferentiation process contributes to aggravation of tumor malignancy (6, 7, 49, 85). As an illustrative example, Bochet *et al.* demonstrated that Wnt3a, which is secreted by breast tumor cells, can induce the conversion of adipocytes into adipose-derived fibroblasts, thereby accelerating breast cancer progression (6). These revelations prompted us to consider the inhibition of adipocyte dedifferentiation as a novel approach for cancer therapy that aims to reduce the aggressiveness of cancer. Recent studies suggest that hindering dedifferentiation mitigates the malignancy of tumors (141). In dedifferentiated LPS cells, induction of apoptosis by irinotecan involves activation of *C/EBPα* (141). *C/EBPα*, a tumor suppressor and transcriptional regulator governing adipocyte differentiation, plays a pivotal role in this context (142, 143). Nonetheless, the precise methods for regulating adipocyte dedifferentiation in these tumor environments and ensuring its safety necessitate further experimental investigations (Fig. 4).

### DFAT cells are an abundant source for stem cell therapy

DFAT cells have emerged as a highly promising source of stem cells for advanced tissue engineering and cell therapy. This is due to their unique characteristics that address some limitations associated with other stem cell sources (144, 145). Firstly, there is an abundant source of DFAT cells. Thanks to the wide distribution of mature adipocytes, DFAT cells are highly abundant in the human body, offering a practically unlimited source of somatic stem cells for research and therapeutic purposes (3). Secondly, DFAT cells are highly homogeneous, which can lead to more predictable and consistent research and transplantation outcomes due to their uniform characteristics (36). Thirdly, DFAT cells possess a strong ability to proliferate, serving as a crucial feature for generating sufficient cells for therapeutic applications (146). Fourthly, with the low risk of tumorigenicity, the transplantation and application of DFAT cells are much safer when compared to embryonic stem cells and induced pluripotent stem cells (43, 114). Overall, DFAT cells have an attractive combination of characteristics that make them a valuable and versatile resource for stem cell therapy and regenerative medicine. Their ability to address the limitations of other stem cell sources, such as ASCs and BMSCs, positions them as a promising avenue for further research and clinical applications (Fig. 4).

### New strategy to improve skin fibrosis and promote wound healing

Scleroderma is a chronic autoimmune disease that can cause severe skin fibrosis and WAT loss (147, 148). Fat transplantation has shown a significant therapeutic effect in treating scleroderma, not only complementing volume loss but also improving skin fibrosis (149, 150). Recently, Wang *et al.* found that fat cells in transplanted fat could migrate to fibrotic skin lesions and undergo dedifferentiation. These DFAT cells could then redifferentiate into fat cells at the lesions, making contributions to the reaccumulated dermal adipose tissue (134). Wang *et al.* also evaluated the therapeutic potential of different adipose-derived cells for scleroderma lesions. They found that dermal thickness and collagen levels were reduced, and blood vessel density increased after DFAT cells treatment, fibrosis-related gene expression in human scleroderma fibroblasts, and promoted M2 macrophage polarization (134). DFAT cell therapy has shown comparable efficacy to ASC therapy in inducing angiogenesis and inhibiting macrophage infiltration in fibrotic skin (134). Therefore, DFAT cell therapy may emerge as a new strategy to improve skin fibrosis.

Recent studies have shown that dWAT can dedifferentiate into fibroblasts during wound healing to meet specific tissue repair and functional needs (151). Skin injury can stimulate dermal adipocytes to rapidly undergo ATGL-dependent triglyceride lipolysis, promoting the release of saturated and monounsaturated fatty acids to the injured site. These released fatty acids increase macrophage infiltration at the wound site and promote vascular reconstruction and wound healing (151, 152). Concurrently, extensive lipolysis depletes the stored lipids in dermal fat cells, enabling these cells to dedifferentiate into fibroblasts and migrate to the wound site (151). Fibroblasts play a crucial role in wound healing. Through mitosis, they proliferate rapidly and secrete a large number of collagen fibers. The regeneration of collagen fibers and the formation of new blood vessels contribute to the development of granulation tissue, which ultimately repairs the wound by contracting and aggregating (153, 154). Additionally, fibroblasts derived from adipocyte dedifferentiation can redifferentiate into adipocytes and may help reprogram disordered fibrous tissue in scars into adipose tissue, thereby improving scar appearance (151, 155). Based on these findings, the dedifferentiation of dWAT during wound healing may enhance the healing process. Therefore, strategies to modulate dWAT dedifferentiation may represent a novel approach to improving wound healing and treating scars. Future studies should aim to elucidate the specific mechanisms by which skin injury initiates lipolysis of dermal adipocytes and how adipocyte-derived fibroblasts migrate to the wound bed.

## Challenges and Limitations

The concept of adipocyte dedifferentiation is not new, but several limitations have hindered progress in this field. The first limitation is a lack of specific gene markers. A major limitation has been the lack of specific gene markers that can clearly define the dedifferentiation status of adipocytes. While genes associated with lipid metabolism are significantly downregulated during adipocyte dedifferentiation, genes related to embryonic stem cells, reprogramming, and lineage differentiation markers are upregulated. However, these genes are not specific indicators of adipocyte dedifferentiation because they are also expressed in mature adipocytes. This lack of specificity makes it challenging to accurately track the adipocytes dedifferentiation process (5, 38, 41, 42). Secondly, there is a difficulty in confirming adipocyte dedifferentiation in vivo. Dedifferentiated adipocytes constitute a limited proportion of adipose tissue in vivo and have the potential to redifferentiate into mature adipocytes. This makes it difficult to observe typical morphological changes associated with dedifferentiated adipocytes in adipose tissue sections. More research on reliable and specific markers needs to be conducted for a convincing adipocyte dedifferentiation tracking (156). Thirdly, where the expelled LDs go still remains ambiguous. While the gradual elimination of LDs has been observed in vitro during ceiling cultures, it is unclear how they are processed in vivo (4, 5, 34). Additionally, the potential impact of expelled lipids on local inflammation levels has not been explored. Recent studies suggest that macroautophagy can selectively sequester and decompose LDs in cells, thereby providing a potential novel mechanism. However, it is important to note that selective macroautophagy is primarily thought to target smaller LDs (157). Thus, further investigation is required to determine whether lipolysis, lipophagy or macroautophagy is the principal pathway responsible for lipid efflux during adipocyte dedifferentiation.

## Conclusions

This review provides an insightful exploration of the molecular changes, genetic modifications, and potential applications of DFAT cells, highlighting exciting prospects in the fields of adipocyte biology and regenerative medicine. Adipocyte dedifferentiation holds promise for a range of applications, from regenerative medicine to cancer therapy and obesity management. Nevertheless, the field faces challenges and limitations that necessitate further research and development. Developing specific markers to track adipocyte dedifferentiation, improving cell purity, and ensuring the safety and efficacy of in vivo applications will be pivotal for advancing this field in the future.

## Data Availability

Data sharing is not applicable to this article as no datasets were generated or analyzed during the current study.

## Conflict of interests

The author declares that they have no conflicts of interest with the contents of this article.
